# ADHD Symptoms as a Stressor Leading to Depressive Symptoms among University Students: The Mediating Role of Perceived Stress between ADHD and Depression

**DOI:** 10.3390/ijerph191711091

**Published:** 2022-09-04

**Authors:** Aida Sahmurova, Selma Arikan, Mustafa Can Gursesli, Mirko Duradoni

**Affiliations:** 1 Faculty of Health Sciences, Antalya Bilim University, Antalya 07190, Turkey; 2 Faculty of Arts and Humanities, Psychology, Istanbul Medeniyet University, Istanbul 34700, Turkey; 3 Department of Education, Literatures, Intercultural Studies, Languages and Psychology, University of Florence, 50135 Florence, Italy; 4 Department of Information Engineering, University of Florence, 50139 Florence, Italy

**Keywords:** Adult Attention Deficit and Hyperactivity Disorder, perceived stress, depression, university students, mediating variable

## Abstract

Attention deficit hyperactivity disorder (ADHD) is a condition manifested in childhood and includes symptoms, such as attention deficit, impulsivity, difficulty in concentrating, hyperactivity, and aggressive behavior. Along with the negative effects of ADHD symptoms on individuals, various psychological factors are thought to be associated with these symptoms. However, ADHD in emerging adulthood is not rare and can be conceived as the continuation of discomfort that might emerge during childhood and adolescence. Our mediation study involved 613 undergraduate students in İstanbul. The participants filled out demographic information forms: Adult Attention Deficit and Hyperactivity Disorder Self-Reported Scale (ASRS), Perceived Stress Scale and Beck Depression Inventory (BDI). Our work stressed those positive correlations found among ASRS, Perceived Stress Scale, and BDI scores. Analysis by Model 4 of Hayes Process Macro and Sobel tests showed that ADHD symptoms predicted both perceived stress and depression scores of the participants and dimensions of perceived stress acted as partial mediators in the positive relationship between ADHD and depression scores.

## 1. Introduction

By negatively affecting the quality of life and the academic performance, psychological problems have always been among the crucial health issues involving university students [1]. Among those psychological problems, depression is one of the most prominent. Several studies have shown that approximately 17–23% of university students experience depression, and this rate goes up to 45% among those students applying to counseling services of the universities [2]. Time spent in university is a critical period as students are transitioning from adolescence to adulthood, a period that has been quite recently defined as emerging adulthood [3]. Thus, it is critical to reveal the factors which are likely to cause depression among university students in order to take the necessary precautions against those factors to provide them with more productive and satisfactory academic lives which in turn will affect and improve their quality of life. Academic achievement was found to be a principal source of stress among university students [2]: in order to achieve success during their undergraduate education, the students must follow their courses regularly, concentrate on and study their course materials carefully, and conduct their assignments accurately. They also must be organized and good at planning so that they can fully carry out the responsibilities they are assigned by their instructors. Time management skills, concentration, and determination are also crucial for academic performance. Thus, for those students affected by attention deficit hyperactivity disorder (ADHD), the route to academic achievement becomes more challenging and complex.

Contrary to the common belief stating that ADHD is a childhood problem, studies have shown that it may also endure in adolescence and adulthood [4]. ADHD in emerging adulthood can be conceived as the result of unsuccessful or absent treatment of the disorder during childhood and adolescence [5]. The prevalence of adult ADHD in the USA is assumed to be approximately 4%, with a good proportion who are not aware they have ADHD [6]. ADHD symptoms may include chronic impulsiveness, inattentiveness, poor concentration, and hyperactivity. ADHD symptoms start in early childhood and may continue throughout one’s life [4]. Some of the symptoms associated with adult ADHD can be listed as short attention span, difficulty in prioritizing tasks, getting bored easily, being easily distracted from the tasks that require reading or attention, and avoiding tasks that are boring or uninteresting. Adults with ADHD may also face problems such as ineffective time management, impulsive decision-making, and difficulties of concentration on tasks. They may fail to accomplish the critical tasks they are expected to do, as they choose to focus on less important ones. Problems related with difficulties in organizing, focusing, and completing tasks may induce stress, anxiety, and feelings of inadequacy among those who experience these problems [4].

The literature showing that the social and academic demands cause stress among university students; thus, the current study aims to examine how adult ADHD symptoms, as a potential source of academic strain, predict stress levels and depressive symptoms of undergraduate students. We also intend to investigate the mediating role of stress on the relationship between ADHD and depressive symptoms.

### 1.1. Adult Attention Deficit and Hyperactivity Disorder

As a neuropsychiatric disorder, ADHD is often associated with childhood. However, recent follow-up studies have shown that between 50% and 70% of ADHD incidences persist into young adulthood [7]. The symptoms of ADHD may diminish with age while some of them may turn into restlessness and uneasiness as the person grows into adolescence. As for adults, the main symptoms of ADHD may include impatience, difficulty in planning, and problems with focusing on tasks [8]. ADHD prevalence rate is found between 5.9 and 7.1% among teenagers and adolescents; while the prevalence rate of adult ADHD is estimated to be between 1.2% and 7.3%. The rate of university students with ADHD varies from 2% to 8% throughout the world [7]. The prevalence of adult ADHD has been the subject of research in our country Turkey, as well. Conducting questionnaires on 1961 university students, Kılıçoğlu et al. [9] reported that 15.55% of their participants showed ADHD symptoms. In their study conducted in Sivas province, Yapıcıoğlu et al. [10] examined 901 people aged between 18 and 44 through questionnaires and clinical interviews. Analysis of the questionnaires revealed that the prevalence of adult ADHD was 3.8%, which turned out to be 2.7% based on the clinical interviews. Similarly, Kavakçı et al. [11] carried out a study with 980 students studying at Cumhuriyet University by use of questionnaires and clinical interviews. The percentage of subjects with adult ADHD was found to be 10% based on questionnaires and 6.1% based on the clinical interviews.

Adults with ADHD suffer from concentration and focusing problems more than they do from hyperactivity and impulsivity. ADHD-related difficulties cause social and academic problems. Concentration problems and difficulties in completing assignments are the common problems observed in the academic lives of those with ADHD. Due to such problems, individuals with ADHD cannot improve their academic and social skills and are subject to the risk of experiencing problems in their adulthood in terms of career, family, and social life. Some psychiatric disorders that are comorbid with ADHD, such as mood disorders, anxiety, and learning disability, can also increase that risk [12].

### 1.2. ADHD and Stress among University Students

Self-reported studies in the literature for ADHD, conducted in different samples show that university students have a significant level of ADHD symptoms. Weyandt et al. [13], clarify in their study of 770 people and a single university sample, showed that 7–8% of the participants had significant ADHD symptoms. In addition, in the study conducted by McKee [14] on 1096 university students, 7% of the participants found the number of symptoms, which is one of the necessary criteria for diagnosis. Moreover, DuPaul et al. [15] another study carried out in three different countries and different universities found the distribution of significant symptoms according to different genders between 0% and 8%. These previous studies in the literature are also another proof of the necessity and importance of the research carried out.

When students start university in their late adolescence, their life becomes more demanding than ever. They may find it hard to adapt to a new academic and social life. Besides, most of them leave their family for the first time in their lives and move to another city alone. Adaptation to a new system, leaving family, the pressure their family exerts on them to succeed, financial problems, academic competition, course load, and exam stress cause excessive stress among the students [16]. Excessive stress may result in physical and psychological problems over time. It has been reported in the literature that loss of appetite, sleeping problems, lack of energy and motivation, headache, and gastrointestinal problems can be observed among those students who have excessive stress [16]. Literature also revealed that stress may not only increase anxiety but also cause weight change, mood swings, problems with work habits, and even suicidal thoughts [17]. Bayram and Bilgel examined stress levels of 1617 university students and found that 27% of the students had stress of moderate level or above [18].

Some of the problems experienced by adolescents or adults with ADHD, such as planning and organizing problems, time management difficulty, and difficulties in setting priorities, focusing, paying attention, and completing tasks can create extra stress [4]. Those kinds of problems reduce academic performance, as well. Low academic performance may further increase academic stress experienced by students. In this respect, we estimate that an increase in ADHD symptoms will increase stress as well.

**H1.**  *Stress scores of university students are expected to increase as their ADHD symptoms increase. In other words, ADHD scores of students predict their stress scores.*

### 1.3. Depression among University Students

Depression is characterized by several symptoms including bad mood, lack of pleasure and interest, interrupted sleep, changes in appetite, fatigue, low energy, poor concentration, indecision, feeling of worthlessness, slowing down, or agitation of physical movements [19,20,21]. The prevalence of depression is estimated to be around 30% among university students, which is quite above the rate estimated for the general population [22]. The results of the research conducted by Bayram and Bilgel revealed that the prevalence of depression among Turkish university students was 27.1% [18]. Moreover, Mikolajczyk et al. found significant levels of depressive symptoms in four European countries’ university students [23]. Additionally, Chen et al. attested that 11.7% of the participants had a BDI score 14 or higher, in their research in Chinese universities with a sample of 5245 [24]. These studies showed that the phenomenon of depressive symptoms affects different countries around the world, and this makes it crucial to investigate the relationship between depressive symptoms along with other variables.

Many studies have been carried out regarding the effect of depression on the academic performance of students and the results unveiled that depression could cause an approximately half-point decrease of GPA (grade point average). It was also found that students missed classes or exams due to depression [25]. Depression is also reported to hinder students’ professional development as they may consider leaving school due to it [26].

### 1.4. ADHD, Stress, and Depression

Stressful situations and life events are triggering factors that can lead to depression. Quite a few studies were conducted on university students regarding the relationship between stress and depression. Dahlin et al. [27] carried out a study on Swiss medical students in which they found that academic workload, future concerns, inadequate feedback, and low commitment, which were some stressors pointed out by the participants, predicted students’ depression scores. Dahlin et al. [27] added that academic stress might also affect cognitive functions and learning skills negatively, which may further deteriorate the problem.

Academic performance is one of the most fundamental qualifying requirements for students to graduate from school [28,29]. There are many factors that affect academic success such as sleep, mood, social dimensions [30,31,32]. One of the most important of these factors is ADHD symptoms. The literature shows that there is a significant relationship between college students with ADHD symptoms and low academic performance [33,34]. Moreover, the relationship between academic performance and depression has been revealed by various studies [35,36]. Considering this and many similar studies carried out, the authors think that ADHD symptoms may predict depression scores.

Adult ADHD-related problems may create extra stress on university students in addition to their academic load and university transition [37]. Excessive stress may bring out depressive symptoms [38]. As a matter of fact, in their research involving 1648 students, Rabiner et al. [39] found that academic stress increased depressive symptoms and they also stated that attention-related problems had an important role in this relationship. Thus, we anticipate a positive correlation between adult ADHD scores and depression scores [40] parallel to the literature as well as between stress scores and depression scores. Besides, ADHD symptoms are expected to increase depression scores through the mediator role of students’ stress levels. In line with our expectations, other hypotheses of our research can be listed as follows:

**H2.**  *Depression scores of university students are expected to increase as their ADHD symptoms increase. In other words, ADHD scores of students predict their depression scores.*

**H3.**  *Depression scores of university students are expected to increase as their stress scores increase. In other words, stress scores of students predict their depression scores.*

**H4.**  *Through increasing their stress levels, ADHD symptoms increase the depression scores of the students. In other words, the stress level acts as a mediator in the positive relationship between ADHD and depression.*

## 2. Method

### 2.1. Sample

The sample of the study consists of 613 students studying at a foundation university located in İstanbul. Among those 613 participants, 234 are males and 379 are females. The average age of males is 21.39 (s.d. = 1.914), while the average age of females is 20.90 (s.d. = 1.634). The data were collected from various faculties’ students: 267 students from the Health Science faculty, 47 students from Social Sciences and Humanities faculty, 87 students from the Engineering faculty, 32 students from the Management Sciences faculty, 71 students from Law School, 24 students from the Education faculty, 26 students from Architecture faculty, 38 students from Applied Sciences faculty, and 21 students from Fine Art faculty. All participant students were volunteers. Demographics are presented in Table 1.

### 2.2. Measures

#### 2.2.1. Demographic Information Form

The participants were asked to fill in a demographic information form, including questions about their gender, age, faculty, department, class, GPA, and familial matters.

#### 2.2.2. Beck Depression Inventory

21-item Beck Depression Inventory was used to identify the depression levels of the participants. Participants were asked to choose one of the four responses, ranked from zero to three, for each item to indicate how they have been feeling in the last week including the day they were asked to respond to the questionnaire. The scale was translated into Turkish by Hisli in 1989 and the validity and reliability of the scale were ensured [41]. For the data collected from 613 participants, the internal reliability analysis was performed, and the Cronbach’s alpha coefficient was found to be 0.89.

#### 2.2.3. Perceived Stress Scale

The 14-item Perceived Stress Scale, developed by Cohen et al., was used to measure participants’ stress levels [42]. The scale was adapted to Turkish by Eskin et al. [43] and the validity and reliability of the scale were also ensured by them. It is a five-point Likert-type scale asking how stressful the participants feel in certain situations. The scale is interpreted under two sub-dimensions both of which include 7 items: Perceived Insufficient Self-Efficacy and Perceived Stress/Distress. The internal consistency of the scale was analyzed by use of the scores collected from 613 students and Cronbach’s alpha coefficient was found 0.76. Cronbach’s alpha values of the subscales were also analyzed, and it was found 0.78 for Perceived Insufficient Self-Efficacy and 0.78 for Perceived Stress/Distress.

#### 2.2.4. Adult Attention Deficit and Hyperactivity Disorder Self-Reported Scale (ASRS)

The scale was developed by Kessler and Üstün in 2004 [44]. It was adapted to Turkish by Doğan et al. [45] There are eighteen items in the scale, nine of which measure Attention Deficit symptoms while the other nine items measure Hyperactivity/Impulsivity symptoms. Reliability analysis showed that the Turkish version of ASRS has a high level of internal consistency (Cronbach’s alpha = 0.88). Cronbach’s alpha coefficients for ‘inattention’ and ‘hyperactivity/impulsivity’ subscales were also high (0.82 and 0.78). Additionally, the correlation coefficients for two-week test-retest reliability among the 50 subjects were high (for total scores, r = 0.85; for two subscales, r = 0.73–0.89) [45].

### 2.3. Procedure

Before the application of the research, ethical approval was obtained from the ethics committee of the university in which the research would be conducted. After the approval was obtained, the students were informed about the research, and questionnaires were delivered to those who volunteered to participate. All participants’ informed consent was gathered at the beginning of the survey. It took about 10 min for each participant to fill in the questionnaires.

### 2.4. Data Analysis

Data gathered from this study was analyzed by using IBM SPSS Statistics 22 program. Pearson’s r correlation coefficients were calculated to find out the relationships between the variables. Besides, the Process Macro Analysis Method version 3.3, developed by Hayes was used to test the research model. Hypotheses were tested by “parallel multiple analysis” model 4 defined by Hayes [46]. This analysis allowed us not only to test the direct and indirect relationship between Adult ADHD symptoms and depression, but also to spot whether sub-dimensions of stress have mediator roles in this relationship.

## 3. Results

### 3.1. Relationships among the Variables

Foremost, the relationship among the variables was measured through Pearson’s r correlation analysis. The findings are given in Table 2. As shown in the table, significant positive relationships were found between total ADHD scores and total perceived stress scores, as well as between the scores obtained from sub-dimensions of the perceived stress scale and the scores obtained from the depression scale. Besides, the attention deficit subdimension of ADHD was found to be significantly correlated with the insufficient self-efficacy sub-dimension of the perceived stress scale. However, no significant correlation was found between impulsivity/hyperactivity subdimension and insufficient self-efficacy scores.

### 3.2. Analysis for Hypothesis Testing

The bootstrapping Process Macro method, which allows multiple mediation models to be tested, was used for testing the mediator roles of the sub-dimensions of the perceived stress variable. In the multivariate relationship model tested, adult ADHD scores were the independent variable and Beck Depression Inventory scores were the dependent variable. Insufficient self-efficacy perception and perception of stress/distress, which are the sub-dimensions of the perceived stress scale, were analyzed as mediating variables. The 95% confidence interval was taken into account to test whether indirect effects were significant in the model.

The findings showed that the overall effect of adult ADHD symptoms on depression scores was significant (*B* = 0.354, *SE* = 0.05, *t* = 7.304, *p* < 0.001). Adult ADHD explains 8% of the variance in depression scores (*R*^2^ = 0.08, *F*_1, 611_ = 53.345, *p* < 0.001). When mediator variables, namely stress sub-dimensions, insufficient self-efficacy perception and stress/distress perception were added to the model, 25% of the variance was explained (*R*^2^ = 0.25, *F*_3, 609_ = 68.06, *p* < 0.001). The effects of adult ADHD scores on depression scores were significant when the effects of mediator variables were controlled (*B* = 0.213, *SE* = 0.04, *t* = 4.66, *p* < 0.001). In the findings of the bootstrapping analysis, it was found that the total of indirect effects that adult ADHD had on depressive symptoms through the perceived stress sub-dimensions was significant (*B* = 0.213, 95% CI = 0.11 and 0.32, *SE* = 0.05). When the mediation effects of the perceived stress sub-dimensions were examined, insufficient self-efficacy perception (*B* = 0.659, 95% CI = 0.51 and 0.82, *SE* = 0.08), stress and discomfort perception (*B* = 0.503, 95% CI = 0.34 and 0.66, *SE* = 0.08) variables were found to partially mediate the relationship between adult ADHD and depressive symptoms significantly, as the confidence intervals do not contain a zero value in between.

**Figure 1:**
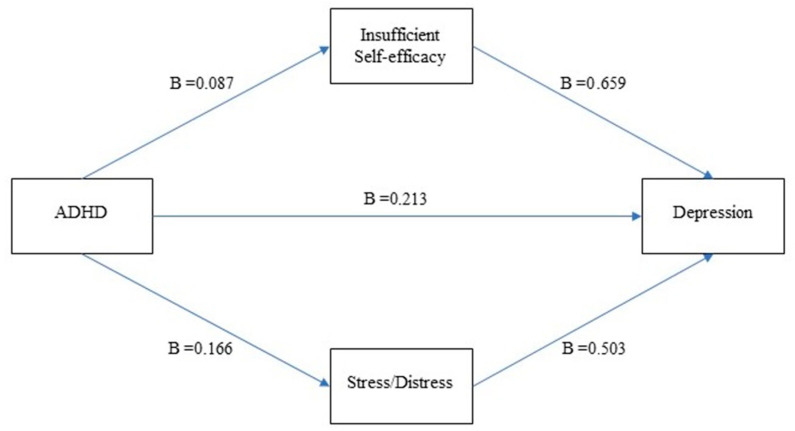
Perceived Stress Dimensions as Mediators in the relationship between ADHD and Depression.

The Sobel Z scores were calculated for the mediating role of perceived stress subdimensions. The mediating roles of perceived insufficient self-efficacy (Sobel Z: 3.399, *p* < 0.001) and perceived stress/distress (Sobel Z: 4.827, *p* < 0.001) were also supported by the Sobel Test. All these findings fully support the first three hypotheses and partially supported the fourth hypothesis we have put forward. Findings regarding the hypothesis testing, including the mediation analysis, are presented in Table 3 and visually represented in Figure 1.

## 4. Discussion

The findings of this study revealed that adult ADHD symptoms can increase the stress levels of university students and can predict depressive symptoms through increased stress levels. Correlation analysis revealed a noteworthy finding, which is the significant positive relationship between the symptoms of adult ADHD regarding attention deficit factor and the insufficient self-efficacy dimension of perceived stress. This finding is in congruence with the previous studies.

Academic performance is one of the most important thing university students focus on in their lives. People around university students also expect them to be successful. Academic performance correlates with variables such as self-esteem and self-efficacy, as well [47,48]. The symptoms of adult ADHD, especially attention deficit related symptoms, may strain university students in terms of their responsibilities as students, and this in turn causes them to feel inadequate. In their study, Rabiner et al. [39] found that ADHD symptoms, especially those related to attention deficit were associated with academic anxiety and depressive symptoms among university students, and this significant relationship endured even when students’ personality traits were controlled. In the study conducted by Shaw-Zirt et al. [49] students diagnosed with ADHD considered themselves less adequate than other students in the academic, social and emotional sense. Likewise, Dooling-Liftin et al. [49] reported that self-esteem was lower in students diagnosed with ADHD.

To sum up, symptoms related to adult ADHD can make students feel inadequate. In their study, conducted on students who have consulted to the university’s counseling services, Heiligenstein et al. [50] eliminated the students with other comorbid diseases and analyzed only those with adult ADHD symptoms. They found that students with ADHD symptoms had lower GPAs than the others. They also reported that the number of students who were on probation was higher among students with ADHD symptoms. There are many other studies in which associations between adult ADHD symptoms and poor academic performance among university students were found.

Our findings regarding the relationship between adult ADHD and perceived insufficient self-efficacy; and the literature showing the relationship between adult ADHD symptoms and academic performance can be evaluated from the perspective of Bandura’s Social Cognitive Motivation Theory [51,52]. The symptoms of adult ADHD, especially those related to attention deficit, cause students to have difficulties in fulfilling their responsibilities, and this condition increases the students’ stress levels and sense of inadequacy and decreases self-efficacy [53,54]. Considering the relationship between low academic performance and self-efficacy in line with the studies in the literature, it is thought that various problems may be observed in the academic life of the students [47,55]. There are also debates in the literature about adult ADHD and university dropouts [56,57]. In this respect, it may be useful for counseling professionals to focus on increasing the feelings of adequacy and self-efficacy levels of the students besides the practices to increase attention and concentration while interventions are conducted for students having attention deficit problems. Still, it is of the utmost importance to carry out holistic research on university students regarding adult ADHD, self-efficacy, academic performance, and social and emotional adaptation based on the Social Cognitive Theory of Motivation.

The findings of our study supported our second hypothesis regarding the positive relationship between adult ADHD symptoms and depressive symptoms, as well. In line with existing literature, our third hypothesis regarding stress predicted depression, has also been supported through our findings [27,58]. Our mediation hypothesis that adult ADHD will predict depressive symptoms through stress was also partially supported. In summary, adult ADHD symptoms can cause stress among students and increase their depressive symptoms for those students who have tendencies to mood disorders.

Future studies should certainly investigate the possible role of ADHD scores as a moderator of the relationship between perceived stress and depression. Moreover, replicating our model by using a different measure of ADHD like clinical interviews with therapists and various ADHD diagnosis instruments would strengthen our findings, as well as including sex and gender dimensions as possible moderators.

Nonetheless, while evaluating the findings, there are several limitations of the current study that should be highlighted. First, we must state that causality cannot be inferred because the research is a cross-sectional one and cross-sectional studies cannot prove causality. It is also evident that the relationships and the variances explaining depression found through the findings are not very high. In other words, there may be many other different factors increasing stress and depression symptoms among students. In addition, the exploratory results of the current study are based on a biased sample because of a non-random sampling method and self-selection bias. Lastly, the use of self-report measures may have resulted in a few possible measurement biases due to the well-known tendency of people to provide socially acceptable responses rather than being truthful (e.g., honesty) and their limited capability of assessing themselves accurately (e.g., introspective ability) [59,60].

Overall, depression may result from various factors. Among those factors, adult ADHD should be taken into consideration; and the mental health professionals providing counseling services at universities should conduct questionnaires and clinical interviews to test whether the students applying for counseling have any ADHD symptoms. Besides, considering the studies reporting that ADHD is an important risk factor for giving up education life, it is important to identify and support those students who have problems regarding adult ADHD [56]. The counseling services may post informative posters and banners on campuses to reach the students that have academic performance problems and scan them for adult ADHD, considering the studies showing that some of the adult ADHD cases are undiagnosed. Systematic support and intervention programs to these students might have the potential to decrease the number of university dropouts caused by low academic performance and a sense of inadequacy. Support programs implemented by Hesslinger et al. [61] in Germany and Nasri et al. [62] in Sweden have achieved significant results. Nasri et al. [62] applied a 14-week intervention program in which they used cognitive behavioral therapy and dialectical behavioral therapy in addition to Hesslinger et al.’s protocol [61]. They found that ADHD symptoms, perceived stress, and depression scores were significantly lower at the end of the intervention. Such interventions should be encouraged in our country Turkey, precisely because it has the potential to enable students with adult ADHD symptoms to have more successful and satisfying academic lives. Such interventions, which will be developed in our country for students with adult ADHD symptoms, will be beneficial for these students as it will enable them to have a more successful and satisfying university education and better careers after graduation.

## 5. Conclusions

Eventually, this work highlighted how ADHD symptoms in emerging adulthood can increase the stress levels of university students and therefore indirectly affect depressive symptomatology. This indirect effect of ADHD on depression is mediated by both perceived stress and perceived insufficient self-efficacy.

## Figures and Tables

**Table 1:** Demographics of the present sample.

Gender	N	Mean Age	Std.	Min. Age	Max. Age				
Male	234	21.39	1.914	18	25				
Female	379	20.60	1.634	18	27				
Total	613	20.90	1.786	18	27				
**Faculty**	Health Sciences	Social Sciences and Humanities	Engineering	Management Sciences	Law School	Education	Architecture	Applied Sciences	Fine Arts
N	267	47	87	32	71	24	26	38	21
**Year**	Prep Year	First	Second	Third	Fourth				
N	13	236	185	104	75				
**Mother’s Education Level**	Unable to Read and Write	Able to Read and Write	Primary Education	Secondary Education	High School Education	Bachelor’s Degree or Higher			
N	10	5	106	90	219	183			
**Father’s Education Level**	Unable to Read and Write	Able to Read and Write	Primary Education	Secondary Education	High School Education	Bachelor’s Degree or Higher			
**N**	3	1	70	78	234	227			
**Accommodation**	Alone	Family	Friends	Dormitory					
**N**	111	289	79	134					

**Table 2:** Correlation Coefficients between ADHD, Stress and Depression Scores.

Variables	1	2	3	4	5	6	7
1. ADHD	1						
2. Attention Deficit	0.88 **	1					
3. Impulsivity/Hyperactivity	0.84 **	0.49 **	1				
4. Total Perceived Stress	0.28 **	0.28 **	0.20 **	1			
5. Insufficient Self-Efficacy	0.15 **	0.21 **	0.05	0.76 **	1		
6. Stress/Distress	0.28 **	0.22 **	0.25 **	0.78 **	0.18 **	1	
7. BDI (Depression)	0.27 **	0.25 **	0.24 **	0.47 **	0.38 **	0.35 **	1

** *p* < 0.01.

**Table 3:** Partial Mediating Role of Stress in the relationship of ADHD and Depression.

Independent Variable	Dependent Variable	*R* ^2^	*F*	*p*	Β	*t*	*p*	95% CI
ADHD	Insufficient Self-Eff.	0.02	13.724	<0.001	0.087	3.704	<0.001	[0.0409, 0.1330]
ADHD	Stress/Distress.	0.07	48.882	<0.001	0.166	6.992	<0.001	[0.1194, 0.2127]
ADHD	Depression	0.08	53.345	<0.001	0.354	7.304	<0.001	[0.2589, 0.4494]
ADHD	Depression	0.25	68.060	<0.001	0.213	4.080	<0.001	[0.1233, 0.3032]
Insufficient Self-Eff.					0.659	8.633	<0.001	[0.5094, 0.8094]
Stress/Distress					0.503	6.668	<0.001	[0.3551, 0.6516]

## Data Availability

The data presented in this study are available on request from the corresponding author.
